# A mixed methods analysis of environmental and household chaos: considerations for early-childhood obesity research

**DOI:** 10.1186/s12889-021-11936-w

**Published:** 2021-10-16

**Authors:** Kathryn L. Krupsky, Andria Parrott, Rebecca Andridge, Bharathi J. Zvara, Sarah A. Keim, Sarah E. Anderson

**Affiliations:** 1 Division of Epidemiology, College of Public Health, The Ohio State University, 336 Cunz Hall, 1841 Neil Ave, Columbus, OH 43210 USA; 2 Center for Biobehavioral Health, Abigail Wexner Research Institute at Nationwide Children’s Hospital, 700 Children’s Dr. NEOB 3rd Floor, Columbus, OH 43205 USA; 3 Division of Biostatistics, College of Public Health, The Ohio State University, 1841 Neil Ave, Columbus, OH 43210 USA; 4 Department of Maternal and Child Health, Gillings School of Public Health, University of North Carolina at Chapel Hill, 135 Dauer Dr., Chapel Hill, North Carolina 27599 USA; 5 Department of Pediatrics, College of Medicine, The Ohio State University, 370 W. 9th Ave, Columbus, OH 43210 USA

**Keywords:** Toddlers, Childhood obesity, Chaos, Mixed methods, Prevention

## Abstract

**Background:**

Chaos has implications for child health that may extend to childhood obesity. Yet, results from studies describing associations between chaos and childhood obesity are mixed. New approaches to studying the environments of young children may help to clarify chaos-obesity relationships.

**Methods:**

We conducted a concurrent mixed methods analysis of quantitative and qualitative data describing home and neighborhood chaos among a diverse cohort of 283 caregiver-toddlers dyads from Ohio. We examined the underlying structure of environmental and household chaos using exploratory factor analysis then sought to validate the structure using qualitative field notes. We generated total scores for factors of chaos and described their distributions overall and according to cohort characteristics. Additionally, we conducted a thematic content analysis of brief ethnographies to provide preliminary construct validity for our indicators of chaos.

**Results:**

Dyads varied according to household composition, income, education, and race/ethnicity. We found evidence for a multi-factor structure for chaos, which included disorganization and neighborhood noise. Household disorganization scores ranged from 0 to 7.3 and were on average 2.1 (SD = 1.8). Neighborhood noise scores ranged from 0 to 4 and were on average 1.1 (SD = 1.1). Both disorganization and neighborhood noise were associated with indicators of socioeconomic disadvantage, such as lower educational attainment and household income. Qualitative data from households with high and low scores on the two identified factors were aligned in ways that were supportive of construct validity and further contextualized the social and material environments in which chaos occurred.

**Conclusions:**

Chaos represents a complex construct with implications spanning various disciplines, including childhood obesity research. Previous studies suggest challenges associated with measuring chaos may limit the conclusions that can be drawn about which aspect of chaos (if any) matter most of early childhood weight development. We advance the literature by demonstrating chaos may be comprised of conceptually distinct subdomains. Future childhood obesity prevention research may benefit from more contemporary measure of chaos, such as those relying on direct observations that account for a multifaceted underlying structure.

## Background

### Childhood obesity

Childhood obesity remains prevalent across the world, presenting one of the most challenging public health problems of this century [1]. Once established, obesity and related comorbid chronic conditions often persist into adolescence and adulthood [2]. The persistent nature of this condition may result from the establishment of obesity-related behaviors in early childhood, including poor diet, obesogenic eating behaviors, and physical inactivity, which often track through adulthood [3]. Therefore, infancy and toddlerhood (birth to 24-months) may be critical periods for obesity prevention efforts.

Health behavior patterns underlying the development of childhood obesity are influenced by a complex ecology of factors [4]. Thus, interventions designed to change obesity-related behaviors require multilevel approaches with multiple components [5]. Currently, no compelling evidence advocates for one program or method for preventing childhood obesity. However, comprehensive approaches addressing both behavioral risk factors and psychosocial support within relevant contexts tend to offer more promising outcomes [6, 7]. For young children, family homes are ideal settings for the implementation of childhood obesity preventions strategies [8–11], as the family home represents a microsystem of processes by which child functioning is shaped [12]. However, more research is needed to understand how features of family homes may influence such efforts.

### Chaos and childhood obesity

Chaos is one feature of family home environments that may be consequential for early-childhood weight development, but evidence is mixed. For example, cross-sectional studies investigating direct associations between chaos and weight status among preschool-aged children report null findings [13, 14]. One prospective study of caregiver-reported chaos, measured twice over a six-month period, indicated higher levels of chaos were associated with higher body mass index (BMI) z-score in among infants [15]. Other studies propose chaos may operate through mediators such as cortisol patterns, child eating behaviors, and sleep duration to contribute to poorer child weight outcomes [14, 16].

Chaotic environments are often described as noisy, crowded, and lacking organization [17]. Furthermore, both structural and temporal instability in the form of frequent changes in adults’ romantic partners, residential mobility, loss of family income, and disrupted family routines and rituals may also contribute to chaos [18]. Chaos is more likely to occur among households with fewer socioeconomic resources [17]. However, transdisciplinary research consistently demonstrates the detrimental effects of chaos on child health and development, above and beyond the influence of socioeconomic status (SES) [17–19], even among samples that are homogeneous, with regards to SES [20]. Moreover, it has been proposed that chaos may act as the mechanism by which SES affects child health and development [20–22].

Within the context of the family home, chaos may undermine essential processes that facilitate healthy weight development [23]. For example, the elevated stimulation and unpredictability associated with higher levels of chaos may be stressful for young children [14]. Additionally, caregivers living in more chaotic households may exhibit less warmth and responsiveness towards their children [24, 25]. It has been proposed that the ways in which caregivers respond to their children during moments of heightened stress may impact the organization of emerging stress physiology in early life [26]. The mechanisms responsible for physiological responses to stress are closely related to the processes governing mood and appetite regulation [27, 28], which are linked to obesogenic child eating behaviors [29, 30]. Moreover, caring for a small child is difficult and can be stressful for caregivers, especially as children reach toddlerhood and demand more autonomy [31]. The rapid development occurring in toddlerhood is often coupled with new behaviors that may add to family-level chaos and create barriers to establishing household structure and routines, which may protect against obesity in early childhood [32]. Thus, examining chaos amidst the unique challenges of toddlerhood may provide critical information that can be used to inform early childhood obesity prevention strategies.

### Measuring Chaos in childhood obesity research

A recent systematic review of chaos and structure in family home environments in relation to early childhood obesity found associations with child weight outcomes in the majority (10 of 14) of studies, but the direction of results was inconsistent, and measures of chaos differed substantially [33]. This variability is likely due, in part, to challenges associated with measuring chaos. Chaos is complex and is thought to be composed of multiple constructs [34], yet most studies examining chaos-obesity relationships refer to only specific indicators of chaos, like crowding [35] or a lack of routines [32, 36]. Conversely, other popular measures, such as the Confusion, Hubbub, and Order Scale (CHAOS) [37], may lack the nuance necessary to identify important subdomains of chaos [34]. Coinciding with this critique, Lumeng and colleagues (2014) used a subset of eight items to describe “emotional chaos” as a component of the CHAOS measure, in their study of stress, eating behaviors, and early-childhood weight outcomes [14]. Furthermore, measures like the CHAOS rely on caregiver perceptions which tend to be subjective and influenced by factors such as coping strategies and personality traits [38].

One potentially promising strategy for measuring family-level chaos may include direct observations of family homes using numerous environmental indicators. To the best of our knowledge, no study has described such a strategy in the childhood obesity literature. However, examples exist in other research domains. For instance, lead investigators of The Family Life Project [39], a study designed to longitudinally assess child health and development and family function among households living in low-income rural regions of the U.S. [39], conducted five direct observations of household chaos over a three year period using 10 indicators (e.g., household moves and household cleanliness) [34]. Factor analyses of these 10 indicators resulted in a two factor structure consisting of disorganization and instability [34]. In subsequent analyses of these chaos findings from the Family Life Project, researchers showed that chaos in the form of disorganization was more important for outcomes like child language development [34] and academic achievement [22], than chaos in the form of instability. Thus, a more objective approach to assessing chaos using multiple chaos-related conditions may support efforts to reconceptualize aspects of chaos and determine which (if any) matter for early-childhood weight outcomes.

### Study aims and objectives

In the present study, we aimed to characterize the home and neighborhood environments of a contemporary cohort of toddlers and explore potential contributors to chaotic environments. Our analyses utilized direct observations of family homes. To accomplish our aim, we examined chaos using a concurrent mixed methods research design. The objectives for this study were (1) to examine the underlying structure of environmental and household chaos using direct observations of the immediate neighborhood and family home environment and (2) to establish preliminary evidence for construct validity using qualitative fieldnotes. We hypothesized that more comprehensive and nuanced assessments of family home environments would provide additional evidence for a multi-factor structure of chaos, including disorganization, noise, and instability. We then discussed our findings in the context of current early childhood obesity literature and offer considerations for future studies.

## Methods

### Study population

Data are from Play & Grow—a prospective cohort study of 299 parent-child dyads from central Ohio. Study design and cohort characteristics for the Play & Grow Study have been previously described [40] but are briefly summarized here. The target population for Play & Grow included 18-month-old children (± 2 months) living in central Ohio. A sampling frame was constructed using patient medical records from Nationwide Children’s Hospital (NCH) in Columbus, Ohio. Caregiver-child dyads were enrolled between 2017 and 2019. Dyads enrolled included primary caregivers (93% biological mothers) and children who were born singleton with gestational ages between 23 and 42 weeks. Enrolled dyads lived within 15 miles of NCH with no family plans to move beyond that radius during the study and the participating caregiver attested to taking part in the child’s meals on a regular basis. Participants were excluded from recruitment if the child had deafness, blindness, food allergies, gestational age > 42 weeks, or if the child was tube-fed or a patient for a clinical feeding disorder. The final cohort consisted of caregiver-child dyads who were diverse with regards to gestational age, race/ethnicity, household composition, education, and household income (Table 1).

**Table 1:** The distribution of participant characteristics from the full Play & Grow cohort compared to participant characteristics of our analytic sample

	Full Cohort (*N* = 299)		Analytic Sample (*N* = 283)		***P*** value
	n	%	n	%	
**Child Characteristics**					
Gestational Age at Birth					
*37–41 Completed Weeks (term)*	188	62.9	181	64.0	0.10
*< 37 Completed Weeks (preterm)*	111	37.1	102	36.0	
Sex					
*Boy*	170	56.9	162	57.2	0.57
*Girl*	129	43.1	121	42.8	
**Primary Respondent Characteristics**					
Age (years) at enrollment					
*18 to < 21*	7	2.3	7	2.5	0.02
*21 to < 25*	46	15.4	45	15.9	
*25 to < 30*	63	21.1	56	19.8	
*30 to < 35*	98	32.8	95	33.6	
*35 to < 40*	62	20.7	59	20.8	
*40 or older*	23	7.7	23	8.1	
Race/Ethnicity					
*Non-Hispanic white*	158	52.8	149	52.7	0.59
*Non-Hispanic Black*	111	37.1	104	36.7	
*Non-Hispanic other (includes multiple races)*	17	5.7	17	6.0	
*Hispanic*	13	4.3	13	4.6	
Educational Attainment					
*High school diploma/GED or less*	70	23.4	62	21.9	0.07
*Some college*	103	34.4	99	35.0	
*Bachelor’s degree*	67	22.4	64	22.6	
*Post-graduate degree*	59	19.7	58	20.5	
Marital Status					
*Single/Never Married*	56	18.7	52	18.4	0.08
*Married*	162	54.2	157	55.5	
*Living with Partner*	64	21.4	57	20.1	
*Other*	17	5.7	17	6.0	
**Household Characteristics**					
Number of Household Members					
*2 members*	16	5.4	15	5.3	0.76
*3 members*	97	32.4	93	32.9	
*4 members*	90	30.1	86	30.4	
*5 or more members*	96	32.1	89	31.4	
Food Insecurity					
*Food Secure*	248	82.9	239	84.5	< 0.01
*Food Insecure*	51	17.1	44	15.5	
Annual Household Income					
*< $20 thousand*	78	26.1	72	25.4	< 0.01
*$20 to < $50 thousand*	89	29.8	79	27.9	
*$50 to < $90 thousand*	57	19.1	57	20.1	
*$90 thousand or more*	73	24.4	73	25.8	
*Missing*	2	0.7	2	0.7	

*Note:* Cohort characteristics were predominately reported by caregivers via survey during the 18-month (enrollment) visit for Play & Grow. *P* values were generated from Chi-square tests. Food insecurity was assessed using the U.S. Department of Agriculture Food and Nutrition Service’s guidelines for measuring food security. We coded families as having food insecurity if they indicated experiencing any level food insecurity in the 12-months prior to them completing our surveys

Play & Grow is ongoing. Thus, we utilized data from the first and second assessments, which took place when children were approximately 18- and 24-months of age. Assessments included caregiver surveys and direct observations of family homes. We limited or analyses to only records with complete data on the variables examined in this study (*n* = 283). Our analytic sample differed from the original Play & Grow cohort, with regards to caregiver age, food insecurity, and household income (Table 1). The study was conducted in accordance with the Declaration of Helsinki and the Institutional Review Board of NCH approved study procedures. Researchers obtained written documentation of informed consent for all subjects.

### Data collection

We utilized Rapid Assessment Procedures (RAP) [41] to simultaneously collect quantitative and qualitative data describing neighborhood and household conditions. RAP make use of traditional anthropological techniques, such as participant observation, interviewing, and analysis of quantitative data, over a shortened and more focused period of fieldwork [41]. Typically, RAP are implemented by multidisciplinary teams across multiple sites, include prompt turn arounds on data analyses, and are participatory in nature [41, 42]. While RAP may never meet the methodological standards sought by most anthropologists, researchers across disciplines increasingly recognize the advantages of RAP for gaining insight into complex social and material settings. For example, RAP have been used in domains including health education [43], pandemic response in clinical settings [44], and health information technologies [45]. Our RAP consisted of quantitative audits of neighborhood and household characteristics and participant observation techniques in neighborhoods and family homes.

#### Observations of neighborhood and household environments

As part of the second wave of data collection, pairs of trained researchers drove to participant homes when children were 24-months of age. Teams consisted of a Lead and an Assistant Researcher. The Lead Researcher directed the visit and led a variety of study protocols. The Assistant Researcher provided support and was tasked with conducting an extensive mixed methods observation of neighborhood and household conditions. To facilitate this observation, we designed a novel data collection tool to describe a variety of neighborhood and household conditions by adapting existing environmental audits and questionnaires [34, 37, 46–48] (Table 2).

**Table 2:** Description of items considered as indicators of neighborhood and household chaos

Item	Indicator	Original Question Stem	Original Response Format	Modified Question Stem	Modified Response Format
1	Interior Noise Rating [34]	House is not overly noisy (e.g., TV, shouts of children, radio, nearby roads or thoroughfares)	No; Yes	How would you rate the amount of noise inside the home? Focus on the noise produced by appliances, people, animals, etc. inside the home	Very quiet; Fairly quiet; Somewhat noisy; Very noisy
2	Hear Exterior Noise Inside [34]	Neighborhood noise around the home	Cannot rate; Very quiet; Average; Noisy; Very noisy	Is noise from outside the home audible when standing inside the home? Is there presence of loud ambient sounds (e.g., trains, construction, factories, traffic, people outside)?	No; Yes
3	Rating of Exterior Noise Audible Inside [34]	Neighborhood noise around the home	Cannot rate; Very quiet; Average; Noisy; Very noisy	How would you rate the amount of outside noise audible from inside the home?	Very quiet; Fairly quiet; Somewhat noisy; Very noisy
4	Noise Pollution [46]	Noise Pollution	None; A little; Some; A lot	Is there presence of **loud** ambient sounds? Can you hear trains, construction, factories, loud engines, etc.?	No; Yes
5	Exterior Noise Rating [49]	Amount of Noise	Very quiet; Fairly quiet; Somewhat noisy; Very noisy	How would you rate the **level of noise** overall?	Very quiet; Fairly quiet; Somewhat noisy; Very noisy
6^*^	Number of Changes to Household Roster	Sum of total changes (e.g., people leaving or joining household) to household roster between 18- and 24-month assessment			No changes; 1 change; 2 or more changes
7^*^	Regular Mealtime Routine [50]	Mealtimes occur at the same time each day			Strongly disagree; Disagree; Neither agree nor disagree; Agree; Strongly agree
8^*^	Number of Adults in the Household	Sum of the total number of adults (age ≥ 18 years) in household at 24-month visit			1 adult; 2 adults; 3 adults; 4 or more adults
9^*^	Number of Children in the Household	Sum of the total number of children (age < 18 years) in household at 24-month visit			1 child; 2 children; 3 children; 4 or more children
10^*^	Caregiver Marital Status Change	During our last visit, you told us that your marital status was: [MARITAL STATUS]. Is this your current marital status or has your marital status changed?			Did not change; Did changed
11^*^	Total Residential Moves	How many times have you moved since [CHILD] was born?	Did not move; Once; Twice; 3 times 4 time; More than 4 times		0 times; 1 time; 2 or more times
12^*^	Regular Bedtime Routine [51]	Do you have a regular routine of things you do with [CHILD] when you put [HIM/HER] to sleep?			No; Yes

13	Cluttered Interior [47]	All visible rooms of the house are reasonably clean and minimally cluttered.		The house is reasonably clean and minimally cluttered	No; Yes
14	Crowded with Furniture [47]	In terms of available floor space, the rooms are not overcrowded with furniture.		Rooms are overcrowded with furniture	No; Yes
15	Commotion [37]	There is very little commotion in our home	Very much like your own home; Somewhat like your own home; A little bit like your own home; Not at all like your own home	There is very little commotion in the home	No; Yes
16	Interruptions [37]	At home we can talk to each other without being interrupted	Very much like your own home; Somewhat like your own home; A little bit like your own home; Not at all like your own home	Family members talk without interrupting one another	No; Yes
17	Preparedness Rating [34]	Home visit preparation by the household	Cannot rate; Surprised/difficulty; Aware but unprepared; Aware/ready; Good hosts	How prepared did the household appear for the home visit?	Not at all prepared; Somewhat prepared; Quite a bit prepared; Very much prepared
18	Loud Speaking [37]	You can’t hear yourself think in our home	Very much like your own home; Somewhat like your own home; A little bit like your own home; Not at all like your own home	Family members speak to one another in an elevated volume	No; Yes
19	Telephone Use [37]	The telephone takes up a lot of our time at home	Very much like your own home; Somewhat like your own home; A little bit like your own home; Not at all like your own home	The telephone took up a lot of time in the home (e.g., ringing or active phone conversations)	No; Yes

20^†^	Arguments [37]	I often get drawn into other people’s arguments at home	Very much like your own home; Somewhat like your own home; A little bit like your own home; Not at all like your own home	Family members are drawn into other people’s arguments in the home	No; Yes
21^†^	Rushed [37]	We almost always seem to be rushed	Very much like your own home; Somewhat like your own home; A little bit like your own home; Not at all like your own home	Family members appear rushed	No; Yes

^*^Items derived from caregiver surveys that were administered as part of the 18- and 24-month assessments

^†^ Excluded from exploratory factor analysis due to little variation in item responses

The Assistant Researcher was also trained to supplement quantitative observation data with a rapid participant observation to describe various conditions and interactions noted in participants’ neighborhoods and homes. Participant observation is a traditional anthropological technique often used when researchers aim to develop an understanding of participants’ lived experiences amidst natural settings [52]. We adapted key features of participant observation to be implemented over numerous home visits lasting approximately 100 minutes each.

For at least 10 min prior to the start of the home visit, the immediate neighborhood (the spaces visible from the participant’s home) was observed from the vehicle with the windows down (weather permitting). The Assistant Researcher also examined the exterior of the family home while unpacking study materials from the vehicle and walking to the entrance of the participant’s home. The interior of family homes was observed for the remainder of the scheduled visit (approximately 90 min). The Assistant Researcher was permitted to interact with participants in ways that built rapport. However, conversations between the study team and participants occurred mostly with the Lead Researcher. This left ample time for the Assistant to observe and discreetly write descriptive fieldnotes. Assistant Researchers were also encouraged to practice critical self-reflexivity by writing notes describing their experiences, challenges, or personal biases they noticed while conducting observations. Following visits, study staff returned to research offices where they logged their neighborhood and household observations and wrote a brief ethnography using their jotted fieldnotes.

### Research staff training and reliability

Prior to data collection, research staff received a half-day training involving a two-hour classroom session (discussing skills and techniques of ethnography [53]) and a two-hour field practice component. A second classroom-based review session was conducted once data collection was underway. Photos of varying neighborhood and household conditions were rated and discussed. Detailed descriptions of each rating were provided. Based on group consensus, definitions for ratings and descriptions were recorded and organized in a manual for reference and future trainings. During the field component, trainees traveled to the home of a research team member where each trainee completed and discussed the observation form. Trainees, who consisted mostly of college-educated, white, middle-class females under age 40 years, were required to demonstrate 80% rater agreement from a minimum of five observations before they were certified to collect data.

### Data analyses

#### Quantitative analysis

We used items from the observation of neighborhood and home environments to describe chaos. The original data collection tool consisted of 32 items. However, some of these items were better indicators of socioeconomic disadvantage or more aligned with constructs such as neighborhood social cohesion [54] or neighborhood disorder and decay [48]. Therefore, we selected 14 items that were most like other measures of environmental and household chaos (Table 2). We also included another seven items derived from caregiver surveys that were administered as part of the 18- and 24-month visits (Table 2) to supplement measurements of household instability (often characterized by changes in parental romantic relationship status, household moves, changes in income or parental employment, and disruption to family routines) [18]. We chose to do this because such indicators are not possible to observe during a 100-min home visit. We reviewed the distributions of responses for the initial 21 items and chose to exclude two due to little variability in item responses. Thus, we sought to empirically derive measures of environmental and household chaos from a total of 19 items (Table 2).

We developed scales describing chaos using exploratory factor analyses (EFA) [55] with unweighted least squares and oblique (Promax) rotation methods. All items considered for the EFA were ordinal or binary. Therefore, our factor analysis was based on polychoric correlations, rather than Pearson’s correlations [56]. We chose to employ unweighted least squares for ordinal indicators, because it has been shown to be robust to smaller sample sizes, skewed data, and provides greater accuracy and less variability in estimates, when compared to diagonally weighted least squares [57].

Factor extraction was informed by a scree plot [58] and our theoretical understanding of chaos. Our final factor structure required factors to have a minimum of three items, as fewer than three items generally results in weak factors [58]. Following previously published work, we assigned an item to a factor if the primary loading was ≥ |0.40| [57] and the item did not cross-load (loading was <|0.32| for other factors) [58]. If an item failed to meet our criteria for factor assignment, the item was removed during the structure development process. Once our factor structure was identified, we generated summary scores for our measures of chaos. Because our EFA included items with different response options (e.g., No = 1, Yes = 2 versus Very Quiet = 1, Quiet = 2, Noisy = 3, Very Noisy = 4), we chose to rescale items within a range [0, 1] using min-max feature scaling [59] to ensure items were equally weighted. Once rescaling was completed, we summed items according to their factor assignments then estimated the internal consistency for each scale using Cronbach’s Alpha.

We conducted descriptive statistics (means, standard deviations (SD), and *P* values from one-way ANOVA) to described how measures of chaos distribute across characteristics of the sample, including child, caregiver, and household characteristics. Variables to describe cohort characteristics were predominantly derived from the caregiver survey administered at the 18-month assessment. Quantitative analyses, including the EFA, were conducted using SAS (version 9.4, SAS Institute, Cary, NC).

#### Qualitative analysis

Due to the large number of homes visited by researchers during the 24-month assessment, we chose to examine and compare qualitative records from a randomly selected subset of families. To do this, we categorized factor scores into quartiles and randomly selected records from the highest quartile and records from the lowest quartile of factor scores. A thematic analysis [60] was conducted using the brief ethnographies. Informed by the indicators of chaos selected from the neighborhood and household observation, we used a deductive approach to develop codes, though an initial round of open coding was completed to assess patterns in the data and codes missing from our a priori coding structure [61]. A final codebook was constructed with code definitions to ensure consistency across coding. Coding was conducted by the first author and records were coded until thematic saturation [52] was achieved. A total of 88 records were coded and codes were managed electronically using *QSR NVivo* (Version 12, QSR International, Victoria, Australia).

## Results

### Quantitative findings

The scree plot showed a large break between two and three factors and a smaller break between four and five factors. The eigenvalues for the first five factors were 4.1, 2.2, 1.5, 1.2, and 0.9. The total variance explained by two, three, and four factors combined were 58.2, 72.4, and 83.4%, respectively. We examined the factor structures of a two, three, and four factor solutions to assess the face validity of each potential scale. Both the three and four factor solutions contained numerous indicators that cross-loaded and factors with less than three items. Therefore, we concluded a two-factor solution provided the best fit for our data.

Our final factor structure required factors to have a minimum of three items, factor loadings ≥ |0.40|, and no cross-loaded items. As a result, seven items were excluded from our factor assignments (Table 3). Eight items were assigned to the first factor. We labeled this factor *household disorganization* and it included items describing interior household conditions and household dynamics, such as interior noise, clutter, commotion, overcrowding with furniture, telephone use, communication between household members, and overall preparedness for the study visit. Our second factor, labeled *neighborhood noise*, consisted of four items describing the types and amount of noise heard outside participants’ homes (Table 3).

**Table 3:** Factor loadings from exploratory factor analysis of environmental and household chaos indicators

		Factor 1:	Factor 2:
Item No.	Item Name	Household Disorganization	Neighborhood Noise
1	Interior Noise Rating	**−0.79**	0.16
13	Cluttered Interior	**0.51**	−0.31
15	Commotion	**0.84**	− 0.04
16	Interruptions	**0.61**	− 0.05
17	Preparedness Rating	**0.78**	−0.11
18	Loud Speaking	**−0.61**	0.09
14	Crowded with Furniture	**−0.40**	0.06
19	Telephone Use	**−0.47**	0.30
2	Hear Exterior Noise Inside	0.08	**0.75**
3	Rating of Exterior Noise Audible Inside	−0.05	**0.84**
5	Exterior Noise Rating	−0.26	**0.65**
4	Loud Ambient Sounds	−0.13	**0.45**
*Items not assigned to any factor*			
6	Number of Changes to Household Roster	−0.20	0.05
7	Regular Mealtime Routine	0.23	−0.02
8	Number of Adults in the Household	0.07	−0.03
9	Number of Children in the Household	−0.26	−0.06
10	Caregiver Marital Status Change	−0.31	−0.11
11	Total Residential Moves	−0.31	0.06
12	Regular Bedtime Routine	0.34	−0.06

*Note*: N = 283; Exploratory factor analysis using unweighted least squares and Promax rotation; Inter-factor correlation was 0.13, *p* = 0.03; Cronbach’s Alpha 0.73 and 0.67 for household disorganization and neighborhood noise, respectively; Items were not assigned to any factor if their primary factor loading was < |0.40| or if they cross-loaded (had a factor loading ≥ |0.32| with another factor)

After rescaling items within a range of [0, 1], theoretical scores for household disorganization could range from [0, 8], though observed scores ranged from [0, 7.3] (mean = 2.1; SD = 1.8) (Table 4). Theoretical and observed scores for neighborhood noise ranged from [0, 4] (mean = 1.6; SD = 1.1). Cronbach’s Alpha was acceptable for both scales (0.73 and 0.67 for household disorganization and neighborhood noise respectively).

**Table 4:** The distribution of household disorganization and neighborhood noise according to cohort characteristics

	Household Disorganization			Neighborhood Noise		
	(Range: 0–7.3)			(Range: 0–4)		
	Mean	SD	*P* Value	Mean	SD	*P* Value
**Overall**	2.1	(1.8)	–	1.6	(1.1)	–
**Child Characteristics**						
Gestational Age at Birth						
*37–41 Completed Weeks (term)*	2.0	(1.8)	0.14	1.5	(1.1)	0.43
*< 37 Completed Weeks (preterm)*	2.3	(1.7)		1.6	(1.0)	
Sex						
*Boy*	2.1	(1.7)	0.97	1.6	(1.1)	0.90
*Girl*	2.1	(1.8)		1.6	(1.1)	
**Primary Respondent Characteristics**						
Age (years) at enrollment						
*18 to < 21*	3.4	(1.7)	< 0.001	2.4	(0.9)	0.02
*21 to < 25*	2.9	(1.7)		2.0	(1.1)	
*25 to < 30*	2.3	(1.9)		1.4	(1.0)	
*30 to < 35*	1.7	(1.6)		1.5	(1.1)	
*35 to < 40*	1.6	(1.8)		1.5	(1.1)	
*40 or older*	2.2	(1.7)		1.3	(0.9)	
Race/Ethnicity						
*Non-Hispanic white*	1.5	(1.6)	< 0.001	1.4	(1.1)	0.01
*Non-Hispanic Black*	2.8	(1.8)		1.8	(1.1)	
*Non-Hispanic other (includes multiple races)*	2.5	(1.7)		1.7	(0.7)	
*Hispanic*	1.6	(1.6)		1.2	(0.9)	
Educational Attainment						
*High school diploma/GED or less*	2.9	(1.6)	< 0.001	2.1	(1.0)	< 0.001
*Some college*	2.7	(1.9)		1.5	(1.1)	
*Bachelor’s degree*	1.2	(1.1)		1.5	(1.1)	
*Post-graduate degree*	1.0	(1.3)		1.3	(1.0)	
Marital Status						
*Single/Never Married*	2.9	(1.8)	< 0.001	1.8	(1.1)	0.02
*Married*	1.5	(1.6)		1.4	(1.1)	
*Living with Partner*	2.7	(1.7)		1.8	(1.0)	
*Other*	2.8	(1.7)		1.5	(1.1)	
Endorsed Symptoms of Depression						
*No*	2.0	(1.8)	0.07	1.6	(1.1)	0.45
*Yes*	2.7	(1.7)		1.7	(1.2)	

**Household Characteristics**						
Housing Type						
*Single Family Home*	1.7	(1.6)	< 0.001	1.4	(1.1)	< 0.001
*Other*	2.8	(1.8)		1.9	(1.0)	
Number of Household Members						
*2 members*	2.7	(1.9)	< 0.001	1.2	(0.8)	0.26
*3 members*	1.5	(1.5)		1.7	(1.0)	
*4 members*	1.8	(1.5)		1.5	(1.1)	
*5 or more members*	2.8	(1.9)		1.5	(1.1)	
Food Insecurity						
*Food Secure*	1.9	(1.7)	< 0.001	1.5	(1.1)	0.05
*Food Insecure*	2.8	(1.7)		1.9	(1.1)	
Annual Household Income						
*< $20 thousand*	3.0	(1.8)	< 0.001	2.0	(1.1)	< 0.001
*$20 to < $50 thousand*	2.5	(1.9)		1.6	(1.0)	
*$50 to < $90 thousand*	1.4	(1.1)		1.2	(1.0)	
*$90 thousand or more*	1.1	(1.3)		1.4	(1.1)	

*Note*: Participant characteristics were predominately derived from caregivers surveys collected at the 18-month (enrollment) assessment for Play & Grow. Descriptions of household disorganization and neighborhood noise were derived from direct observations conducted in family homes when children were approximately 24-months of age. Higher scores of household disorganization and neighborhood noise are indicative of higher levels of chaos; SD = standard deviation; *P* values were calculated using one-way ANOVA; Food insecurity was assessed using the U.S. Department of Agriculture Food and Nutrition Service’s guidelines for measuring food security. We coded families as having food insecurity if they indicated experiencing any level food insecurity in the 12-months prior to them completing our surveys. Depression symptomology was determined using the Center for Epidemiological Studies Depression Survey, with a score ≥ 16 indicated symptoms of depression

Children’s gestational age and sex were not associated with measures of chaos (Table 4). As expected, higher levels of both household disorganization and neighborhood noise were associated with characteristics often indicative of socioeconomic (dis)advantage, though there was variability within these groups. Caregivers living in more disorganized homes and more noisy neighborhoods tended to be younger, identify as non-Hispanic Black or non-Hispanic other (including multiple races), were less educated, and were more often unmarried. Additionally, participants from more disorganized homes and noisy neighborhoods less often lived in single-family homes and reported lower annual household income. The number of household occupants was associated with household disorganization, but not neighborhood noise, with higher ratings of disorganization assigned to households with two occupants or five or more occupants and lower ratings assigned to households with three or four occupants. Symptoms of depression were marginally associated with household disorganization (*p* = 0.07), but not associated with neighborhood noise (*p* = 0.45). Finally, food insecurity was significantly associated with more household disorganization (*p* <  0.001), but only marginally associated with neighborhood noise (*p* = 0.05).

### Qualitative findings

#### Household disorganization

Qualitative data from our brief ethnographies supported the quantitative indicators that were selected to describe household disorganization (Table 5). The indicator for commotion provided a summary of the amount of movement, activities, and noise occurring within the home. Thus, other indicators, like the interior noise rating, interruptions, and loud speaking, were often intertwined with descriptions of commotion within households. In homes with higher levels of disorganization (quartile 4), researchers often noted people talking over one another or yelling. Staff also noted increased foot traffic within homes. For example, one observer wrote,

*“…several people… were coming in and out of the house while we were there… a teenage boy present at the beginning of the visit…left. The… father also came home partially through the meal… people were entering and exiting from… the back of the apartment.*”

In contrast, homes labeled as being the least disorganized (quartile 1) were depicted as *“…not having a lot of activity going on.”* Observers used words like *“calm”*, *“peaceful”*, and *“relaxing”* to describe to overall atmosphere for such homes and family members were often described as speaking at low, even volumes (some staff used the phrase “*indoor voices”*). Qualitative descriptions of telephone use also varied. Namely, in households that were the most disorganized, observers more frequently described noise from telephones or use of mobile phones during visit activities, whereas in the most organized homes, staff noted rarely seeing or hearing mobile phones.

**Table 5:** Example excerpts providing preliminary construct validity of indicators of chaos selected from the exploratory factor analysis

Quantitative Indicators	Levels of Chaos	
	**High Disorganization**	**Low Disorganization**
(15) Commotion	With the limited space, siblings going up and down stairs, parents going up and down stairs and elevated voices, it felt very chaotic in the home.	The home was calm and peaceful...Both parents… did not appear to be in any rush….. [The] home [did] not have a lot of activity going on.
(1) Interior Noise Rating	…there were six other siblings that were in the home two of which were very young and stayed in the kitchen with staff, they all spoke in their normal speaking voices, but with the small space it echoed loudly in the home and seemed louder… The smoke detector beeped during the entire visit indicating the battery needed to be changed and the washer was running during... activities.	The home was mostly quiet. The child whined..., [but] was not overly loud. Dad and sister played and read together. [They] giggled and spoke but were mostly quiet/[indoor] speaking volume. I could not hear any sounds from household appliances.
(16) Interruptions	A few minutes into the visit, multiple siblings and friends walked through the back door. They loudly spoke over one another, and it was difficult to hear [the] mom and [child] over them. Throughout the visit, mom would yell across the house at siblings to get them to do various tasks or to come join for the meal.	While family spoke often, their voices were never raised. I would describe it as using “indoor voices”.
(18) Loud Speaking	The great grandma got upset with the dogs [for barking] and yelled at them several times saying “I’m going to bust you!”	They never raised their voices to the children but were stern [at times]…
(19) Telephone Use	Mom’s phone made a lot of noise… Mom spent most of our visit looking at her phone and playing with the youngest baby.	I never heard or saw mom or dad use their phone during the visit.
(13) Cluttered Interior;	..The seven-year-old [cousin]… described losing her stuff in the home due to how cluttered it is. She [said she] carried her toys and clothes around in a trash bag to keep track of them.	Playroom had lots of interactive toys: cars with racetrack that could shoot off the cars into a loop-de-loop, play kitchen set, animated toys that sing/dance, blocks/building things. Toys were scattered around the edges of the room, or in smaller baskets, so it seemed like the space had an organization system.

(14) Crowded with Furniture	When we walked into the home, there was a living room with a couch, big chair, two desk chairs, highchair, coffee table, tv stand, and TV. In addition to the furniture, there was a guitar, big speakers, random pieces of wood, unidentified electronic devices, and lots of wires. This room was hard to navigate, and I had trouble finding a spot to do anthropometric measurements. I also felt like I kept accidentally bumping into furniture or knick-knacks on the furniture.	The living room had a couple very large couches and a round leather ottoman and coffee table, but it was not overcrowded and there was a lot of open space.... Against the back wall were many plastic bins with kids toys organized in them, and some toys out on the floor.
(17) Preparedness Rating	Survey was done hours before the visit... [When we arrived] grandma answered the door and seemed surprised we were there. [She] spent at least 3 min wrangling the dogs to get them out of the way while we waited outside.	Dad greeted us at the door promptly after we knocked. The dog was locked away upstairs before we even entered... Dad showed signs of remembering details of the study and seemed prepared for what we were doing at the home visit... Mom was running a few minutes late, but she wasn’t the primary respondent and dad had already communicated that she was be home a few minutes after we arrived prior to us showing up for the home visit. All signs indicated the family was well-prepared for our visit.
	**High Neighborhood Noise**	**Low Neighborhood Noise**
(5) Exterior Noise Rating	The apartment complex... was in was on the corner of a fairly busy cross section. About 20 cars passed by in front of the home and about the same amount passed by on the other direction to the side of the building... We heard a emergency vehicle siren and honking, most likely more than one for about 3 mins or more... Occasionally heard a low rumbling of a large engine in the distance. Around the same time as the sirens, we heard a small engine airplane and then a helicopter nearby. When all was quiet, there was a faint whoosh from the traffic in the distance.	The neighborhood was very quiet. I could not hear any highway traffic, only the occasional car driving past where we were parked or a nearby street. Traffic volume was light. I only counted 4–5 cars passing by during the observation. I did not note any noise from airplanes or trains. Neighbors from across the street were walking out to their car and yelling. One person yelled, “Oh my God! Unlock the door”.

(4) Loud Ambient Sounds	In the 15 mins we sat there, 3 airplanes went by producing a very loud sound and lasting for at least a min each time.	...the sounds of a very high altitude plane flying overhead--it was so faint that I wouldn’t consider it “loud ambient” noise.
(2) Hear Exterior Noise Inside	When sitting in the living room you can hear the cars/trucks passing by from the street. Also hear people’s voices outside, one airplane sound, and music (possibly coming from a different apartment but also may be outside). [I rated it] as very noisy because of the multiple sounds.	I was not able to hear any noise coming from outside, even when we were hanging around the front door to hide out of site during the meal
(3) Rating of Exterior Noise Audible Inside	...the sound from the road traffic outside was very noticeable inside the home. The adult’s bedroom shared the wall that faced the street, and so did the main living space. Child’s room was towards the back of the house. I could hear regular passenger vehicles, but especially large semi’s as they drove past.	I was only able to hear some of the traffic from outside when we were hiding out of sight by the front door.

Descriptions of clutter inside homes were often interwoven with descriptions of crowding due to furniture. Together, these indicators appeared to illustrate the organization of a home’s physical space. For example, in homes that were the most disorganized, staff illustrated environments where tables and other surfaces were covered in various items, like household ornaments, papers, laundry, or appliances. During one home visit, a household member shared her experience of *“losing her stuff in the home due to how cluttered it [was]”*. Staff also described environments that were so crowded with furniture and other items that the space appeared to be *“unusable”* or *“difficult to get around”*. In contrast, homes described as having the least disorganization appeared to have organizational systems in the form of *“bins”* or *“shelves”* that helped *“consolidate”* household items, like children’s toys. Additionally, furniture did not appear to interfere with mobility throughout the home.

The rating for family preparedness often reflected signs that participants remembered their scheduled visit and attempted to follow visit-preparation instructions provided by the research team. In homes with high levels of disorganization, observers frequently noted that caregivers did not complete surveys ahead of time and did not place pets in a separate space before allowing staff to enter their home. Additionally, other family members sometimes appeared *“surprised”* by the presence of staff, suggesting participants did not inform them of the visit. Conversely, staff were often greeted promptly by caregivers from homes with the least disorganization. Family members could sometimes be seen waiting at the door or looking through windows in anticipation of study staff arrival. Caregivers often complete surveys within the recommended timeframe and placed family pets in separate rooms or outside prior to staff entering the home. There was also evidence from the least chaotic homes of family communication in preparation for the visit. For example, one observer wrote, *“…It was evident that mom and dad had discussed the study… before we got there… [because] there was no question about who would do what activities…”*.

In addition to themes directly supporting our quantitative measure of household disorganization, we also noted excerpts describing aspects of the social environment that extended past our eight indicators. For example, in the least disorganized homes, observers consistently noted exchanges and interactions between household members that were generally peaceful and harmonious. In such cases, both caregivers and children appeared to be *“very engaged”* and enjoyed their time together. One observer wrote,

*“[The child] was engag[ed] and seem[ed] really happy to be with mom. Mom… always responded to him, was chatting with him, and smiling at him. [The child] often looked to mom for feedback… Even when mom was busy with the survey, she was always paying attention to [the child] enough to [respond] to him when he [addressed] her.”*

Peaceful exchanges noted by staff often occurred during home visits where more than one caregiver was present (in most cases the child’s mom and dad). Caregivers were described as behaving *“warm[ly]”* and *“friendly”* towards one another and were often successful at *“divide… tasks between each other*”. Caregivers’ ability to remain *“calm and patient*” with their children, even during tantrums or moments of fussiness, appeared to result in more peaceful interactions overall.

Staff also noted displays of affection or support between family members living in more organized households. Such descriptions often included moments when researchers observed a caregiver and child hugging or cuddling. Caregivers shared with study staff positive feelings they had towards their children. For example, one father *“… spoke about [his children’s] school and about the [child participant’s] growth in vocabulary and mentioned that he had counted to eight.”* In other instances, staff noted other family members, like siblings, demonstrating support for the participating child,

*“[older sister] wanted to help [her brother] when he was upset during measurements by showing him how to do them [and] that they were easy and not at all scary. She even held his hand during height measurements...”.*

In contrast, homes with higher levels of disorganization often included staff descriptions of more turbulent exchanges between household members. In such cases, caregivers and children were often observed struggling through visit activities.

*“Mom… seemed very stressed when child did not understand [or] did not follow her requests… [She] kept apologizing throughout the visit… [and] would make… comments [to her son] like, “Come on, you can do this. I know you can do this for us. Why aren't you?”’...”*

In fewer cases, staff witnessed family members engaging in active arguments during the study visit. For example, one researcher observed, *“Mom and partner [having] another hushed, but agitated argument when they thought we were out of ear shot. Mom used several expletives.”* Finally, researchers often used words like *“passive”* or *“lack of engagement”* to describe caregivers in households that were more disorganized.

#### Neighborhood noise

Qualitative data from our brief ethnographies provided preliminary construct validity for our indicators of neighborhood noise (Table 5). In most neighborhoods, the exterior noise rating represented a holistic description of the types of noise and the volume of such noises observed over a brief period. Across levels of neighborhood noise, staff indicated car traffic, in the neighborhood or on a nearby thoroughfare, was a consistent source of noise. However, among a subset of neighborhoods with the highest noise ratings, observers highlighted the home’s proximity to major highways/interstates. One staff member wrote,

*“The apartment complex was built directly next to the highway. There was only a concrete partition separating the highway from the complex parking lot. The partition did very little to cut down noise. It sounded like I should be able to see the vehicles as they drove past, but I couldn't. The highway noise was extremely loud and constant. I could identify every semi-truck that went by.”*

Conversely, in the quietest neighborhoods, observers would sometimes draw attention to the distance between the participant’s home and known interstate highways (e.g., *“the neighborhood is a little more secluded from the interstate and main road”*). In such cases, staff sometimes noted a participant’s home was in *“a [more] rural part of the city”*, on a cul-de-sac, or had a *“dense forest of trees blocking…”* sounds from nearby highways.

Ambient noises were often the same across neighborhoods, despite the level of noise observed. Airplanes and emergency vehicle sirens were frequently heard. Occasionally, staff would note the presences of noise from a nearby construction site or an individual vehicle with a loud engine. However, among homes described as having the lowest neighborhood noise, researchers more frequently described sources of ambient noise as being noticeable to a lesser degree. They did this by conditioning their descriptions using words like *“faint”* or *“muffled”*, or described noise as being *“sporadic”* rather than *“constant”*. Unique to neighborhoods with the highest noise ratings, some observers noted the presence of loud music playing from vehicles driving through the neighborhood. One researcher wrote, *“two cars pulled in at different times that had their music turned up very loud… we could feel [vibrations from] the bass”*.

The ability to hear neighborhood noises from inside participant homes differed according to neighborhood noise ratings. In homes from the quietest neighborhoods, research staff often made statements like, *“no outside noises [could be] heard from inside the home”*. Sometimes staff would suggest that interior sounds, such as subtle humming from appliances or family members talking, might *“drown… out”* the exterior noises. In the few cases where neighborhood noises could be heard while inside the home, observers made a point to note that the family had the *“… windows [and] front door… open because of the weather”* or that study staff *“…were hanging around the front door…”* when they heard such noises. In contrast, while inside homes located in the noisiest neighborhoods, observers repeatedly described hearing vehicle or highway noise and loud ambient noises, such as sirens, airplanes, and at times, construction. For example, one observer wrote, *“We could also hear noise from the highway and construction outside, especially the large trucks going by.”* Another offered, “*[I]* c*ould hear some loud engines revving outside and then an airplane and train at one point.”* In fewer cases, research staff also noted being able to hear loud music playing from passing vehicles while inside the home. For instance, *“…there were several… cars [with loud stereos] that pulled into the complex that we could hear the beat and the tune of the song they were listening to [from] inside the home”*. On the rare occasion that observers were unable to hear neighborhood noises while inside a participant’s home, staff suggested the inability to hear such noises may be due to characteristics of the home. For example, one researcher wrote, *“I tried very hard to hear highway noise and couldn’t. Either the building materials did a good job insulating against noise pollution, or the [air conditioning] was… loud enough… to cover the exterior noise, or both.”*

## Discussion

### Summary of findings

Chaos is known to negatively influence a wide range of family, caregiver, and child outcomes [19]. In recent years, researchers have considered the role of chaos in child weight status, but results are mixed, and conclusions are limited by the heterogeneity in which chaos is operationalized [15, 33, 62]. To improve upon limitations of previous chaos-obesity investigations, we proposed reconceptualizing aspects of chaos to facilitate future research efforts that aim to determine which (if any) matter for childhood obesity development. Thus, in the present study we examined the underlying structure of multiple indicators of family-level chaos from direct observations of neighborhood and home environments, using a concurrent mixed methods approach. We found evidence to suggest numerous indicators of chaos may be governed by higher-order constructs, including household disorganization and neighborhood noise. This evidence was further supported by themes derived from qualitative fieldnotes, which provide preliminary construct validity for a novel chaos assessment tool.

The methodology and results in the present study closely align with those described by Vernon-Feagans and colleagues (2012), who assessed family-level chaos via direct observations using multiple indicators of chaos [34]. Concurrent with their methods and results, we utilized direct observations of family homes and found evidence to suggest chaos may be comprised of multiple constructs. However, our study incorporated both quantitative and qualitative assessment of chaos, which presented opportunities for data triangulation, expansion of descriptions of chaos, and further contextualized the broader social and material environments in which chaos occurred [52]. For example, in addition to providing evidence to support our indicators of chaos, themes emerged from our qualitative analysis to suggest family dynamics varied according to the level of household disorganization. Specifically, among homes with the lowest levels of disorganization, study staff described what appeared to be more peaceful and harmonious exchanges between household members. In contrast, observers noted a greater frequency of turbulent exchanges between household members from homes with the highest levels of disorganization. Previous research suggests greater family functioning [63] and high quality relationships between caregivers and children [64–66] may be protective against childhood obesity. Yet, chaos may degrade the quality of such relationships [24, 67]. It has also been proposed that the quality of interpersonal interactions occurring between family members may moderate the effect of chaos on children’s risk for obesity. For example, one qualitative study conducted semi-structured interviews with 20 ethnically diverse caregiver-child dyads (children aged 9–15 years) and described experiences where household chaos not only influenced the structure and quality of family meals, but also created more challenging mealtime interactions among family members experiencing difficulties in their interpersonal relationships [68]. Similar findings were echoed from one quantitative study among 108 caregiver-toddler dyads where researchers reported children exposed to higher levels of chaos engaged in obesogenic eating behaviors to a greater degree, but only when maternal emotional responsiveness during mealtimes was low [69]. The pathways by which chaos may be linked to child weight outcomes are likely complex, and the unique challenges associated with caring for young children may add to family-level chaos, creating additional stress for caregivers. Our qualitative findings build upon emerging literature which suggests caregiver-child interactions may be important context for studies of chaos-obesity relationships, especially during early developmental periods, like toddlerhood. However, given the exploratory nature of these findings, this interpretation is speculative and requires additional study to determine how chaos and caregiver-toddler interaction may work in concert to influence childhood obesity risk.

Our scale describing disorganization closely aligns with what Matheny and colleagues labeled *environmental confusion*, in the development of the CHAOS [37]. This suggests the CHAOS may provide a foundation for developing measurement tools designed for structured, direct observations of disorganization in family homes. We believe direct observations may be necessary to avoid potential bias often associated with caregiver-reported measures [70]. For example, one study examining parent and adolescent perceptions of household chaos using the CHAOS (*N* = 261 parent-adolescent dyads) found perceptions of chaos in shared home environments were only moderately correlated (r = 0.32), implying individual differences may influence perceptions of chaos [71]. Another analysis examined associations between maternal personality characteristics and perceptions of chaos using the CHAOS (*N* = 94) and concluded mothers with high stimulus sensitivity perceived home environments as more chaotic than what was objectively measured by trained observers [38]. While caregiver-reported measures offer quick, cost-effective alternatives to direct observations, disentangling caregiver characteristics from measures of chaos may be impossible without more objective assessments. Still, direct observations conducted by trained researchers are not without shortcomings, including vulnerability to bias resulting from observers’ personality, knowledge, beliefs, and experiences. We were mindful of this limitation when designing our data collection procedures. To mitigate potential bias in our direct observations, staff were trained to collect both descriptive and reflecting fieldnotes which facilitated staff engagement in reflexivity as they assigned ratings. Never-the-less, individual biases may have played a role in our observations. Therefore, concurrent use of caregiver-reported and objective measures of chaos in future studies may be an essential next step to inform future chaos-related research.

A second factor of chaos, neighborhood noise, was identified through our EFA. Studies examining noise, as one feature of chaos, suggest cardiovascular stress indicators and neuroendocrine stress hormones, implicated in obesity development, may be sensitive to louder environments [72]. However, current definitions of chaos include little specificity around types and sources of noise [17, 37]. Interestingly, one indicator of chaos from our observation, that described the level of interior noise, was highly correlated with our factor of disorganization, but minimally correlated with our factor of neighborhood noise. Such distinctions may suggest noise typologies are a necessary level of nuance for measuring environmental and household chaos, with different implications for childhood obesity research. For example, our thematic analysis further contextualized our neighborhood noise ratings by indicating that participants living in the noisiest neighborhoods often lived close to interstate highways. Highway construction in the U.S. has disproportionately burdened Black and Brown communities and contributed to the residential concentration of poverty [73]. Obesity is known to be a condition that disproportionately affects non-white children [74, 75] and children residing in households with fewer socioeconomic resources [76]. Thus, neighborhood noise may be one aspect of chaos more closely linked with structural disadvantage and requires multifaceted interventions designed to address a variety of upstream social inequalities. Future studies of chaos, within the context of child weight development, may benefit from efforts to describe the variability of chaos within and across socioeconomic groups.

Contrary to what we hypothesized, our EFA did not identify instability as an independent factor contributing to chaos. Prior to selecting our final model, we considered other factor structures that incorporated indicators thought to be associated with instability, such as mealtime routines, bedtime routines, and changes to the household composition. However, the internal consistencies associated with these alternative structures were poor, the face validity was less convincing, and we found little to no support for alternative structures in our qualitative data. Moreover, our lack of support for instability as a factor of chaos may be attributed to the cross-sectional nature of our study design. For example, Vernon-Feagans and colleagues (2012) identified instability as an independent factor of chaos through direct observations of family homes. However, Vernon-Feagans et al. (2012) conducted repeated assessments over a three year period [34]. Unlike other aspects of chaos, which tend to persist, instability often occurs periodically. Thus, single assessments of chaos may be insufficient for detecting factors, like instability. Within this vein, time may be a key component missing in most studies examining associations between chaos and early-childhood weight outcomes. For example, null findings have been reported in cross-sectional studies examining direct associations between caregiver-reported chaos and early-childhood weight outcomes [13, 14]. Conversely, one study that assessed caregiver-reported chaos twice over a six month period indicated infant BMI z-score was significantly higher when household chaos (the average from the two assessments) was higher [15]. Therefore, longitudinal assessments may be necessary for identifying molar constructs thought to govern chaos, as well as associations that may exist between chaos and early-childhood weight outcomes.

### Limitations

Our study has limitations that must be considered. Observations of chaos were conducted during a single visit in participant homes. As some aspects of chaos may be acute while others are chronic, we may not have observed the true variation of environmental and household chaos. Furthermore, the presence of study staff and execution of study protocols during the visit may have contributed to an unusual home environment that factored into our staff’s ratings. Future studies incorporating objective measure of chaos should strive for repeated assessments to ensure what is measured is “typical” for households. Our data collection tool for measuring household chaos was novel. Thus, without more rigorous testing of psychometric properties, construct validity and generalizability of our tool may be limited. Furthermore, protocols for conducting observations were developed and implemented by mostly middle-class, white females and our initial training for our observation protocol was limited to a single field practice component in a neighborhood where one research team member resided. While few studies have examined challenges to conducting direct observations of chaos, a wealth of research demonstrates divergent descriptions of parenting styles when researchers seek to observe behaviors across diverse groups [77, 78]. Though our protocols and trainings attempted to overcome systematic error using multiple methodologies, our homogenous research team did not reflect the diverse cohort of families making up the Play & Grow study. Therefore, it is possible that researcher observations reflect cultural differences that may or may not be symbolic of chaos. Furthermore, most observers interacted and built rapport with families at previous assessments. It is unknown whether these previous interactions influence ratings and fieldnotes. Finally, though we included two items on household routines in our quantitative assessment, household routines were largely neglected from our observations of chaos. Family routines may be key aspects of chaos with important implications for childhood obesity [32, 79]. Future research should combine factors, such as disorganization and noise, with measure of family routines to understand how best to operationalize chaos.

## Conclusions

Chaos represents a complex, multifaceted construct with implications spanning various research disciplines [80], including public health research focused on early childhood obesity. Previous research investigating chaos-obesity relationships in early-childhood may be limited by the challenges associated with measuring chaos. Therefore, this study advanced the literature by contributing to efforts to reconceptualize aspects of chaos and identifying conceptually distinct subdomains, including disorganization and neighborhood noise. As obesity prevention researchers look to family home environments as preferred settings for prevention efforts [81], more contemporary measures, such as those relying on direct observations which account for multiple underlying factors of chaos, may yield valuable insight on factors contributing to early-childhood obesity risk.

## Data Availability

The datasets used and/or analyzed during the current study are available from the corresponding author on reasonable request.

## Abbreviations

CHAOS: The Confusion, Hubbub, and Order Scale
NCH: Nationwide Children’s Hospital
RAP: Rapid Assessment Procedures
EFA: Exploratory Factor Analysis
